# Integration of volatile and non-volatile metabolites and the transcriptome reveals the formation mechanisms of differential aroma compounds between *Pyrus communis* and *Pyrus pyrifolia* cultivars

**DOI:** 10.3389/fpls.2025.1559012

**Published:** 2025-04-02

**Authors:** Jiao Wang, Xianping Guo, Zhongying Wu, Dongsheng Wang, Peng Guo, Yongping Han, Hui Jiang, Zhenzhen Lü

**Affiliations:** ^1^ Institute of Horticulture, Henan Academy of Agricultural Sciences, Zhengzhou, China; ^2^ Henan Horticulture and Floriculture Engineering Research Center, Zhengzhou, China

**Keywords:** aroma compounds, volatile metabolite, transcriptome, pear fruit, synthesis pathways

## Abstract

**Introduction:**

Aroma compounds are important flavor components in pear fruit. Among cultivated pears, fruits from *Pyrus communis* (hereafter referred to as *P. communis*) cultivars are famous for their abundant aroma, while the fruits of most *Pyrus pyrifolia* (hereafter referred to as *P. pyrifolia*) cultivars lack aroma compounds. A comparative study on the formation of differential aroma compounds between the two species could provide a theoretical foundation for improving the aroma quality of *P. pyrifolia* cultivars. However, there is a lack of systematic research on this subject.

**Methods:**

An analysis of volatile and non-volatile metabolites was combined with transcriptome analysis to explore the formation mechanism of differential aroma compounds between three *P. communis* and three *P. pyrifolia* cultivars.

**Results:**

In this study, a total of 510 volatile compounds were identified in the six cultivars. Of these, sixteen ester and alcohol compounds, including butyl acetate, hexyl acetate, ethyl-2-methylbutyrate, ethanol, butanol, propanol, and 2-methylbutanol, with higher contents in the *P. communis* cultivars than in the *P. pyrifolia* cultivars were identified as the primary differential aroma compounds. Among the possible synthesis pathways for these 16 aroma compounds, certain amino acid degradation processes, including isoleucine, valine, and alanine oxidation and threonine dehydration, were found to provide important intermediate substances for synthesis. Within the key enzyme genes in the synthesis pathway, several critical enzyme genes, including monoacylglycerol lipase (PcMAGL, pycom08g09340), threonine dehydrase (PcTD, pycom12g10020), and acyl CoA dehydrogenase (PcACD, pycom16g13880), might be important factors contributing to the disparity in aromatic compounds between *P. communis* and *P. pyrifolia* cultivars.

**Discussion:**

The aforementioned results provide valuable information into the formation mechanisms of differential aroma compounds and offer novel target sites for enhancing pear aroma quality through gene editing.

## Introduction

Aroma compounds are small molecular compounds generated during metabolic processes in plant roots, stems, leaves, flowers, fruits, and other organs. They typically exhibit lipophilic properties and can be released from plants via cell membranes (Pichersky et al., 2006). Aroma compounds in fruit are crucial constituents of fruit flavor and significantly contribute to the assessment of fruit quality (Arimura et al., 2001). They are typically involved in multiple metabolic pathways, which can be categorized into the following three categories based on variations in precursor substances: fatty acid oxidation pathways, amino acid oxidation pathways, and terpenoid synthesis pathways.

Fatty acid oxidation pathways encompass the lipoxygenase (LOX) and β-oxidation pathways. The former uses unsaturated fatty acids as substrates, undergoing a series of enzymatic reactions to produce C6 aldehydes, alcohols, and C9 aldehydes, alcohols, and their corresponding esters (Schwab et al., 2008; Matsui, 2006). For example, aroma compounds such as hexal, hexyl alcohol, hexenal, hexenol, and hexenyl ester, which are found in fruits such as peach, melon, strawberry, and banana, are synthesized through this metabolic pathway. Key enzymes involved in this process include LOX, hydroperoxide lyase (HPL), ethanol dehydrogenase (ADH), and alcohol acyltransferase (AAT) (Beekwilder et al., 2004; Zhang et al., 2010; Chen et al., 2016).

β-oxidation pathways use saturated fatty acids as substrates, which undergo a series of catalytic reactions, including oxidation, hydration, dehydrogenation, and thiolysis, to yield acetyl CoA and fatty acyl CoA. These compounds serve as essential precursors for straight-chain ester synthesis (Napora-Wijata et al., 2014). Furthermore, this pathway provides the primary source for the biosynthesis of volatile substances, such as γ-decalactone, δ-valerolactone, and γ-caprolactone (Xi et al., 2012; Zhang et al., 2017).

A significant portion of volatile aroma compounds, such as alcohols, aldehydes, and esters with a low carbon atomic number, are synthesized via amino acid oxidation pathways (Knudsen et al., 2006). These pathways are responsible for the synthesis of aroma compounds such as 3-methyl-1-butanol, 3-methylbutyl ester, 3-methylbutyric acid, and eugenol methyl ether in banana (Jordán et al., 2001), eugenol methyl ether in strawberry (Aragüez et al., 2013), and eugenol and cinnamate in melon (Gonda et al., 2010). The key enzymes involved in these pathways primarily include aminotransferase (ATF), pyruvate decarboxylase (PDC), alcohol acyltransferase (AAT), and aromatic amino acid aminotransferase (ArAT).

Terpenoids are compounds characterized by an isoprene unit (C5H8) as their fundamental skeleton and serve as crucial constituents of plant aroma substances (Zeng et al., 2020). Grape and citrus fruits exhibit high levels of terpenoids, including s-linalool, which is primarily synthesized through this pathway (Asikin et al., 2012; Yue et al., 2021). The key enzymes involved in this process include 3-hydroxy-3-methylglutarate monoacyl CoA reductase (HMGR), 1-deoxy-D-xylulose-5-phosphate synthetase (DXS), and terpene synthetase (TPS).

Pear (*Pyrus*), which belong to the Pomoideae in the family Rosaceae, originated in the western or southwestern mountains of China during the Tertiary period (Wu et al., 2018). Throughout their dispersal, over thirty recognized species have emerged, with five being most extensively cultivated: *Pyrus pyrifolia*, *Pyrus bretschneideri*, *Pyrus sinkiangensis*, *Pyrus communis*, and *Pyrus ussuriensis* (Li and Zhang, 2020). Most *P. pyrifolia* and *P. bretschneideri* cultivars exhibit either no aroma or a weak aroma (Li and Zhang, 2020; Taiti et al., 2017). However, the *Pyrus sinkiangensis* cultivar ‘Korla’ has a distinct aroma (Tian et al., 2014), while *P. communis* and *P. ussuriensis* develop a pronounced aroma after ripening and softening (Chen et al., 2018; Qin et al., 2012).

Research on fruit aroma in *Pyrus ussuriensis* is primarily focused on ‘Nanguo’ pear. The predominant fruit aroma compounds consist of esters, aldehydes, alcohols, and ketones, with esters being the most abundant (Wei et al., 2016). Ethyl hexanoate, hexyl acetate, and ethyl butanoate are representative aroma compounds found in ‘Nanguo’ pear, and they are predominantly synthesized in the fatty acid oxidation pathway (Li et al., 2022; Shi et al., 2019). In this biosynthesis pathway, the high expression levels of *PuFAD2*, *PuLOX2*, *PuLOX5*, and *PuAAT* are significantly associated with elevated ester concentrations in ‘Nanguo’ pear fruit (Wei et al., 2016; Li et al., 2022). Following treatment with ethylene, low temperature, and methyl jasmonate, significant changes have been observed in aroma compounds and key genes involved in the fatty acid oxidation pathway (Yao et al., 2022; Wu et al., 2023; Shi et al., 2019).

Volatile compounds, including methyl acetate, ethyl acetate, propyl acetate, ethyl dimethylbutyrate, hexanol, and ethanol, are found in high concentrations in *P. communis* pears and are the major contributors to their fruity aroma (Wang M, et al., 2019; Kahle et al., 2005). The contents of these compounds can be reduced through long-term low-temperature storage and melatonin and 1-methylcyclopropene treatment, which may be directly correlated with the decreased activities of LOX, HPL, and AAT in fatty acid oxidation pathways (Makkumrai et al., 2014; Liu et al., 2019; Rizzolo et al., 2005). Ethyl acetate, ethyl butyrate, ethyl acetate butyrate, and hexal have been detected at a significant level in many ‘Korla’ pears, suggesting that these compounds contribute to the characteristic aroma of these pears (Tian et al., 2014).

‘Nanguo’, *P. communis*, and ‘Korla’ pears have been well studied for their aroma characteristics, providing a foundation for further research on pear fruity aroma. However, there is a lack of systematic research on the characteristics and metabolic pathways of differential aroma between aromatic and non-aromatic pear cultivars. In this study, three *P. communis* and three *P. pyrifolia* cultivars were used as materials, and an analysis of their volatile and non-volatile metabolites was combined with transcriptome analysis to investigate the formation mechanisms of differential aroma compounds in the two species. This will establish a theoretical foundation for enhancing the aroma quality of *P. pyrifolia* cultivars and enable further research on the mechanism of aroma formation in *P*. *communis* cultivars.

## Materials and methods

### Plant materials

**Figure 1:**
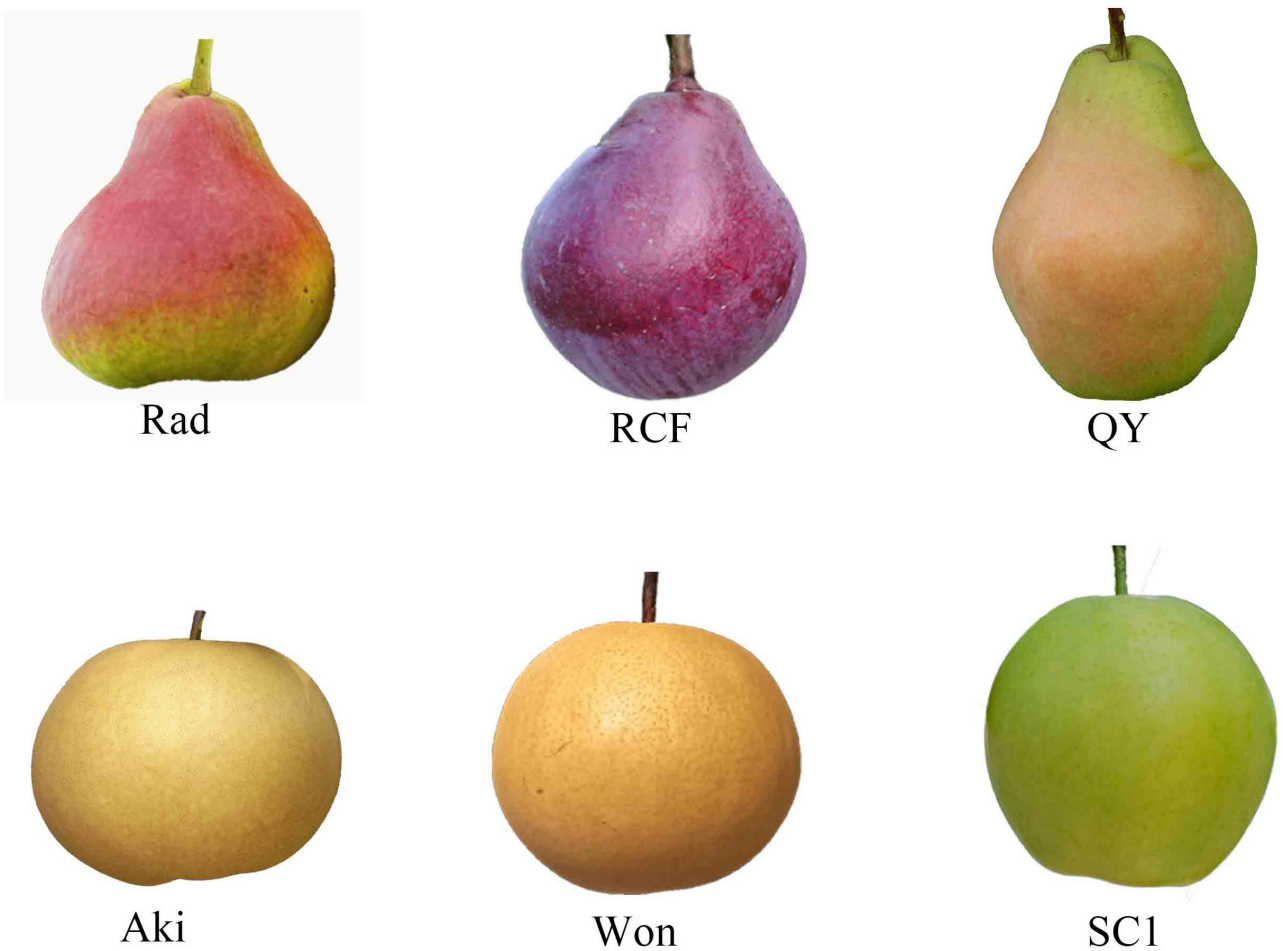
Fruit photographs of 6 pear cultivars for volatile compound determination. The top three belong to the *P. communis* cultivars, while the bottom three are *P. pyrifolia* cultivars.

A total of six varieties, comprising three *P. communis* cultivars (‘Radana’, ‘Red Clapp’s Favorite’, and ‘Qiu Yang’) and three *P. pyrifolia* cultivars (‘Akizuki’, ‘Wonhwang’, and ‘Sucui No.1’), were used to determine volatile compounds (**Figure 1**). A total of 10 varieties, comprising five *P. communis* cultivars (‘Abate Fetel’, ‘Red Clapp’s Favorite’, ‘Qiu Yang’, ‘Radana’, and ‘Packham’s Triumph’) and five *P. pyrifolia* cultivars (‘Akizuki’, ‘Wonhwang’, ‘Whasan’, ‘Mansoo’, and ‘Sucui No.1’), were utilized to investigate the expression levels of key genes in the biosynthesis pathway. For simplification, ‘Radana’, ‘Red Clapp’s Favorite’, ‘Qiu Yang’, ‘Abate Fetel’, ‘Packham’s Triumph’, ‘Akizuki’, ‘Wonhwang’, and ‘Sucui No.1’, ‘Whasan’, and ‘Mansoo’ were abbreviated as ‘Rad’, ‘RCF’, ‘QY’, ‘AF’, ‘PT’, ‘Aki’, ‘Won’, ‘SC1’, ‘Wha’, and ‘Man’, respectively. ‘Rad’, ‘RCF’, and ‘QY’ fruits were harvested at 120, 110, and 160 days after pollination, respectively. They were then left at room temperature for 2 weeks. Once the flesh began to soften, the fruit was cut into small cubes and transferred to an ultra-low temperature refrigerator. The ‘Aki’, ‘Won’, and ‘SC1’ fruits were harvested at 160, 130, and 100 days after pollination, respectively. These fruits were also stored at room temperature for 2 weeks before the flesh was collected. Days after pollination were determined using conventional indices, such as seed color, amylum content, peel color and fruit size (Chen et al., 2018). At fruit maturity, ten fruits were collected from each cultivar. The fruits were peeled, cored, and sliced, after which the sliced flesh from the ten fruits was mixed for volatile compound analysis and transcriptome sequencing.These cultivars were planted at the Yuanyang Test Base of the Henan Academy of Agricultural Sciences, Henan Province, China.

### Volatile compound analysis

The headspace solid-phase microextraction (HS-SPME) technique was employed for the collection of volatile compounds from the fruit of the six cultivars analyzed. Specifically, 2 g of fruit flesh was weighed and placed in a clean vial, which was then sealed with a lid. The sample was agitated at 250 rpm and maintained at 50°C for 15 min and then extracted at 55°C for 30 min. A 50/30 μm DVB/CAR/PDMS fiber (Supelco, Sigma, USA) was utilized for volatile substance extraction from the fruit sample, followed by desorption at 25°C for 5 min to facilitate subsequent gas chromatography–mass spectrometry analysis (Man et al., 2023).

A 7890B gas chromatography system, 5977B mass spectrometry system, and DB-Wax column (30 m × 0.25 mm × 0.25 μm, Agilent Technologies, CA, USA) were employed to detect desorbed volatile compounds. The volatile compounds of the fruit were separated using a constant flow of helium gas at a rate of 1 mL/min, and these separated compounds were then directed to the column. The inlet temperature was set to 260°C. The heating procedure commenced with an initial temperature of 40°C for 5 min, followed by a gradual increase to 220°C at a rate of 5°C/min and further elevation to 250°C at a rate of 20°C/min for 2.5 min. The mass spectra was recorded at 70 eV in electron impact ionization (EI) mode. The ion source temperature was set to 230°C, and the quadrupole mass detector temperature was maintained at 150°C. A comprehensive scanning method was employed to fully analyze the mass spectra of volatile compounds within a scanning range of 20–400 m/z. The chromatogram of each flavor compound was acquired using the LECO Pegasus BT GC-TOF-MS system. The NIST 2017 database was employed to annotate the raw data to obtain the relevant information including the name, retention time, CAS number, and peak area for each compound. A total of 2uL of internal standard (2-octanol) solution were injected into the vial using a syringe.

The content of the volatile compound was determined using the following formula (Wang C, et al., 2019).


$$\text{Content }\left(\text{ug}/\text{g}\right)=\frac{\frac{\text{S}1}{\text{S}2}\times \text{C}2\times \text{V}2\times 10^{-3}}{\text{m}}$$

S1: Peak area of the volatile compound.

S2: Peak area of the internal standard (2-octanol)

C2: Concentration of the internal standard (40ug/mL)

V2: Volume of the internal standard (2uL)

m: Quality of the fruit flesh (g)

OAV were calculated according to the following formula (Nuzzi et al., 2008):


$${\text{OAV}}_{\text{i}}=\frac{{\text{C}}_{\text{i}}}{{\text{OT}}_{\text{i}}}$$

Ci: Concentration of the volatile compound i in the fruit flesh.

OTi: Odor threshold concentration of the volatile Compound i.

### Non-volatile compound analysis

Thermo Vanquish (Thermo Fisher Scientific, MA, USA) was employed for liquid chromatography analysis. An ACQUITY UPLC^®^ HSS T3 (2.1 × 100 mm, 1.8 µm) column (Waters, Milford, MA, USA) was utilized with a flow rate of 0.3 mL/min at a column temperature of 40°C and a sample size of 2 μL. In the LC-ESI (+)-MS mode, the mobile phase consisted of 0.1% formic acid in acetonitrile (v/v) (B2) and 0.1% formic acid in water (v/v) (A2). The elution procedure was as follows: 0–1 min, 8% B2; 1–8 min, 8–98% B2; 8–10 min, 98% B2; 10–10.1 min, 98–8% B2; 10.1–12 min, 8% B2. In LC-ESI (-)-MS mode, the mobile phase consisted of acetonitrile (B3) and ammonium formate (5 mM) (A3). The elution procedure was as follows: 0–1 min, 8% B3; 1–8 min, 8–98% B3; 8–10 min, 98% B3; 10–10.1 min, 98–8% B3; 10.1–12 min, 8% B3.

A Thermo Orbitrap Exploris 120 (Thermo Fisher Scientific, USA) with an electrospray ionization (ESI) ion source was utilized for mass spectrometric detection. Simultaneous MS1 and MS/MS (Full MS-ddMS2 mode, data-dependent MS/MS) acquisition were employed. The spray voltages ESI(+) and ESI(−) were set at 3.50 kV and −2.50 kV, respectively. The sheath gas pressure was maintained at 40 arb, and the aux gas flow remained steady at 10 arb. The capillary temperature was set at 325°C. The HCD technique was employed for secondary fragmentation, with a normalized collision energy of 30% and MS2 resolving power of 15,000 FWHM. The initial four ions within the signal were subjected to fragmentation, while superfluous MS/MS data were eliminated through automatic dynamic exclusion (Zelena et al., 2009; Want et al., 2013).

### RNA extraction and sequencing analysis

Approximately 0.2 g fruit flesh was used for RNA extraction. Total RNA was extracted using a kit (Waryong, Beijing, China), treated with RNase-free DNAase (Takara, Dalian, China). NanoDrop 2000 (Thermo, Waltham, MA, USA) was used to measure the oligonucleotide concentration. Purity could be given from the A260/A280 and A260/A230 values and the Agilent Bioanalyzer 2100 Bioanalyzer (Agilent Technologies, Santa Clara, CA, USA).

Magnetic beads with Oligo (dT)s were used to enrich mRNA from 5 mg of total RNA. The mRNA was randomly fragmented with fragmentation buffer, and first-strand cDNA was synthesized with random hexamers (TIANGEN, Beijing, China). Double-stranded cDNA was then synthesized with dNTPs, RNase H, and DNA polymerase I. The double-stranded cDNA was enriched by adding poly-(A)s and PCR amplification. The enriched cDNA was linked to a vector, which was used to construct a sequencing library and analyzed using the Agilent Bioanalyzer 2100 system (Agilent Technologies, CA, USA). cDNA library sequencing was performed in a HiSeq 2500 system (Illumina, San Diego, CA, USA). All of the pear fruit flesh sequencing was performed with three biological replicates. The low-quality reads were removed, and the high-quality data were aligned to the peach reference genome (Bartlett DH Genome v2.0) with TopHat2 using its default parameters. The gene expression levels were calculated as fragments per kilobase per million reads (FPKM). Differentially expressed genes were selected according to FPKM > 1 and fold change (Rad vs/SC1) > 1.5 or< 0.5 (Wang et al., 2022).

### RT-qPCR analysis

RNA was extracted from fruit flesh using a rapid extraction kit (Aidlab, Beijing, China). Approximately 1.5 μg of the RNA was reverse-transcribed into cDNA using the FastKing RT Kit (with gDNASE) (TIANGEN, Beijing, China). The 20-μL PCR reaction system contained 2.0 μL cDNA diluted 10 times by adding ddH_2_O, 0.5 μL forward primer, 0.5 μL reverse primer, 7 μL ddH_2_O and 10 μL SYBR premix (Vazyme, Nanjing, China). Details of the amplification program were listed in **Supplementary Table S12**. The amplification program was run in the LightCycler^®^ 480 II real-time fluorescent quantitative PCR instrument (Roche, BSL, CH). A pear tubulin gene was selected as a control (Wei et al., 2016). The primers used in this study were shown in **Supplementary Table S13**. We utilized the Primer-BLAST tool from the NCBI (National Center for Biotechnology Information) (https://www.ncbi.nlm.nih.gov/) database to design the primers. The amplification efficiency was obtained by referring to the formula E=−1 + 10^-1/S^, with minor modification in the calculation (Pfaffl, 2001). E denoted the efficiency of qPCR, while S indicated the slope derived from the standard curve. cDNA mixture from ten cultivars (as detailed in the Plant materials section) was utilized as templates to evaluate primer amplification efficiency. The relative expression levels of genes were quantified using the 2^ –ΔΔCt method as described by Livak and Schmittgen (2001). Specifically, the mean ΔCt value for a certain gene was first determined. The ΔΔCt value was then calculated by subtracting ΔCt of other gene from the mean ΔCt of the certain gene. Finally, the relative gene expression levels were derived using the formula Power (2, –ΔΔCt).

### Statistical analysis

The pheatmap package in R (v3.3.2) was used to conduct hierarchical cluster analysis (HCA) of volatile compounds. Principal component analysis (PCA) and Kyoto Encyclopedia of Genes and Genomes (KEGG) enrichment analysis of nonvolatile compounds and transcription products were performed using the Omicshare online tool (https://www.omicshare.com/tools/). Analysis of variance (ANOVA) was employed to assess the differences in gene expression across various cultivars, utilizing SPSS statistical software (SPSS 19.0, IBM) for the statistical analysis. Differences were denoted by lowercase letters (e.g., a, b, c, d) (Gai, 2000). P-values for volatile and non-volatile metabolites and transcriptome analysis, as well as the relative expression levels of genes, were calculated utilizing Microsoft Excel 2007.

## Results

### Analysis of volatile compounds in the flesh of *P. communis* and *P. pyrifolia* cultivars

**Figure 2:**
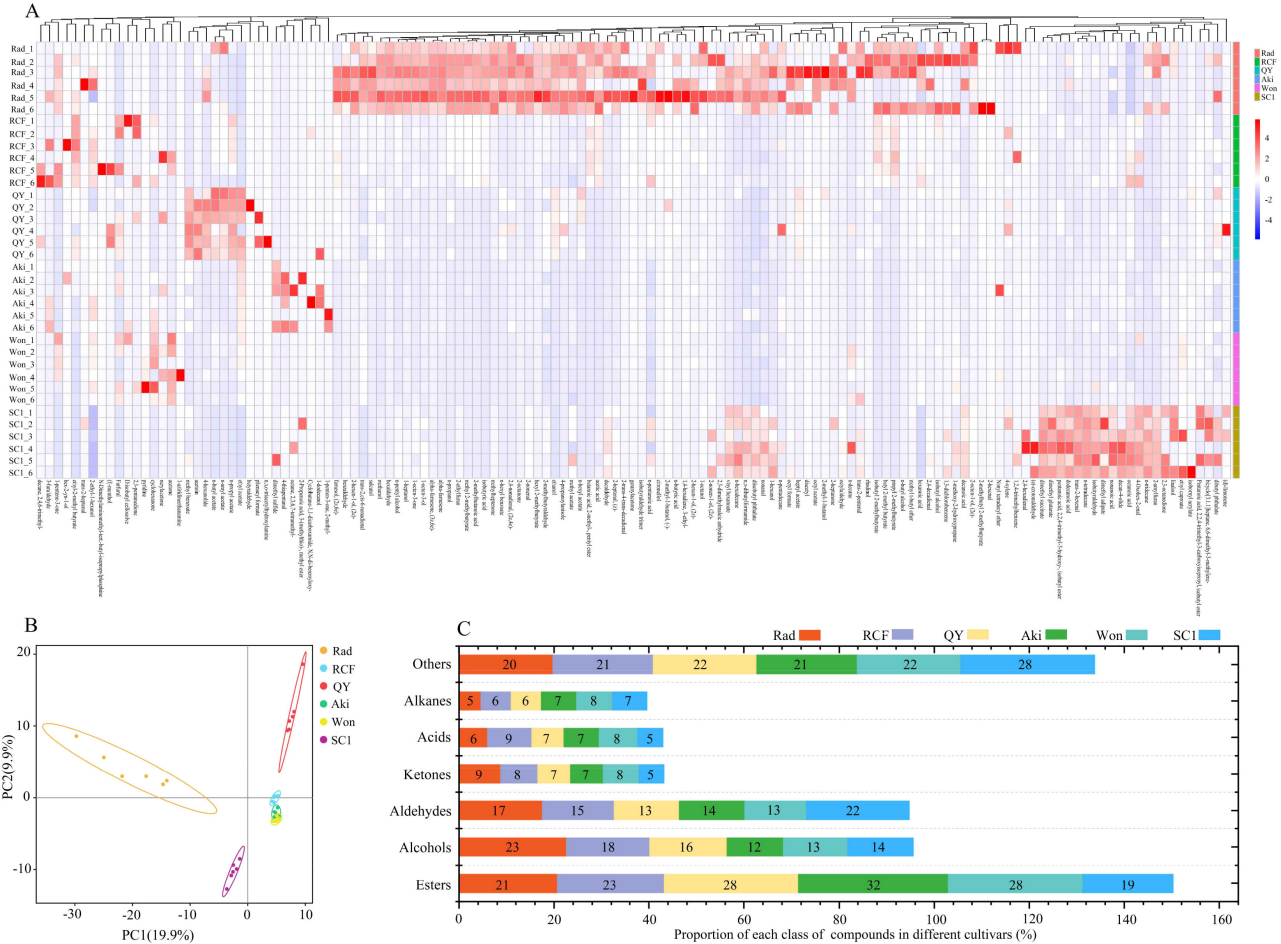
Analysis of volatile compounds in the flesh of six pear cultivars. **(A)** Hierarchical cluster analysis (HCA), **(B)** principal component analysis (PCA), and **(C)** chemical classification of volatile compounds in six pear cultivars.

The volatile compounds in the flesh of three *P. communis* and three *P. pyrifolia* cultivars were analyzed using HS-SPME and gas chromatography. A total of 510 volatile compounds were detected in 6 cultivars (**Supplementary Table S1**), with the following constituting the predominant constituents: esters (Rad (21%), RCF (23%), QY (28%), Aki (32%), Won (28%), and SC1 (19%)), alcohols (Rad (23%), RCF (18%), QY (16%), Aki (12%), Won (13%), and SC1 (14%)), and aldehydes (Rad (17%), RCF (15%), QY (13%), Aki (14%), Won (13%), and SC1 (22%)) (**Figure 2C**). The highest proportion in the three *P. communis* cultivars was comprised of esters and alcohols, consistent with the detection results in the flesh of ‘Conference’ and ‘Packham’ (two *P. communis* cultivars) (Torregrosa et al., 2019; Wang et al., 2019). However, a notable difference was observed in three *P. pyrifolia* cultivars, where esters and aldehydes constituted the predominant components, consistent with the detection results in ‘Atago’, ‘Niitaka’, ‘Banndainiitaka’, and ‘Jinqiu’ (Zhong et al., 2008). These findings suggest that the difference in alcohol and aldehyde levels between *P. communis* and *P. pyrifolia* cultivars contributes to the divergence in their aroma profiles. Subsequently, we conducted HCA on the shared volatile compounds among the six cultivars (**Figure 2A**), revealing distinct components unique to each cultivar. PCA of the volatile compounds also differentiated the three *P. communis* cultivars from the *P. pyrifolia* cultivars (**Figure 2B**). This suggests that volatile compounds among the three *P. communis* and three *P. pyrifolia* cultivars were significantly different and suitable for the subsequent identification of distinct aromatic compounds.

### Identification of the key differentiating aroma compounds in the flesh of *P. communis* and *P. pyrifolia* cultivars

**Figure 3:**
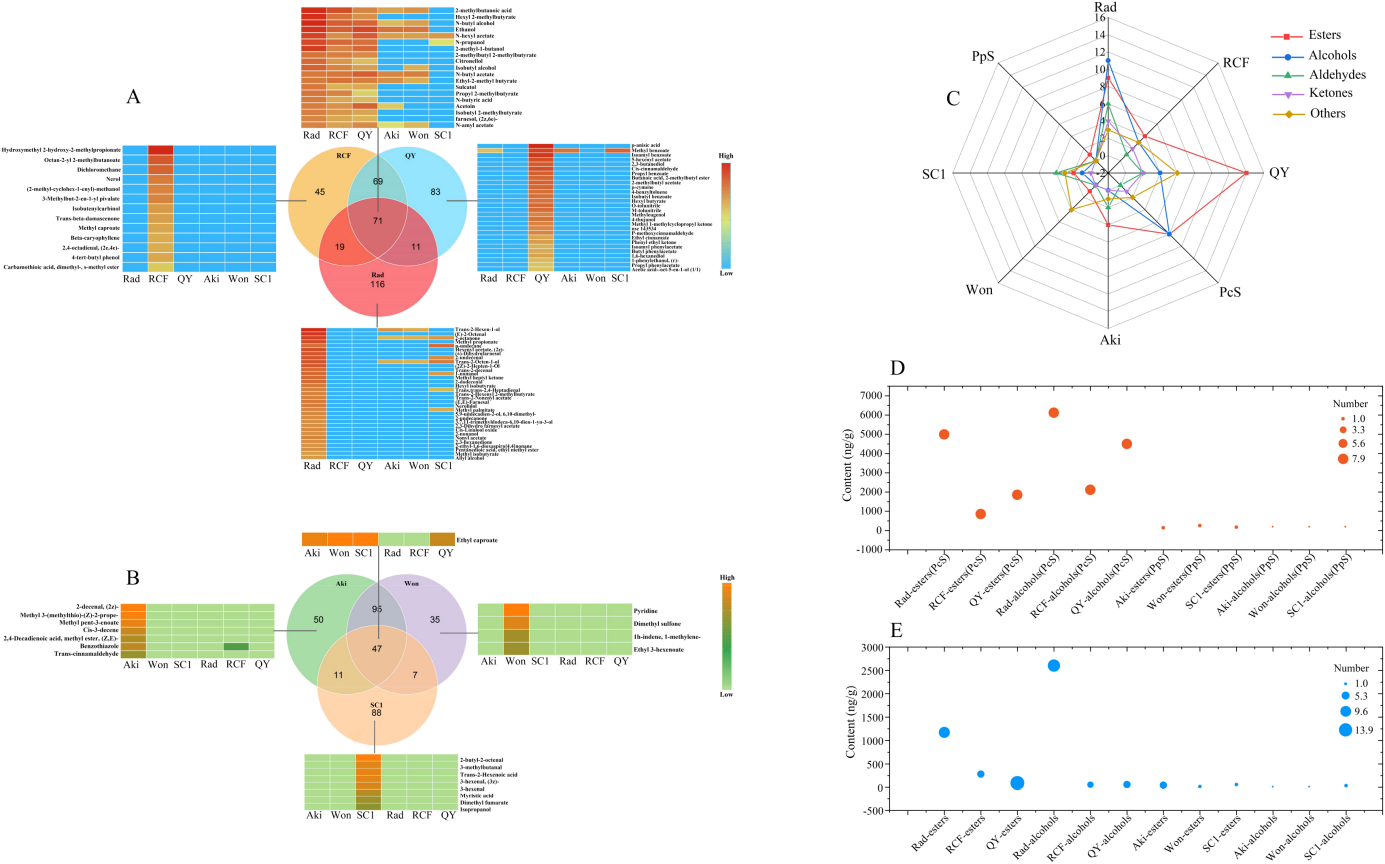
Determination of volatile compounds differing between three *P. communis* and three *P. pyrifolia* cultivars. **(A)** Venn and heat diagrams of common and unique volatile compounds in three *P. communis* and **(B)** three *P. pyrifolia* cultivars. **(C)** Radar map illustrating the distribution characteristics of esters, alcohols, aldehydes, ketones, and other constituents of common and unique volatile compounds found in three *P. communis* and three *P. pyrifolia* cultivars. The ‘common volatile compounds’ herein refer to the constituents that are present in three *P. communis* or three *P. pyrifolia* cultivars, with their concentrations markedly exceeding those found in three *P. pyrifolia* or three *P. communis* cultivars. The ‘unique volatile compounds’ are only expressed in one of three *P. communis* or three *P. pyrifolia* cultivars and constitute the predominant components in six cultivars. ‘Rad’, ‘RCF’, ‘QY’, ‘Aki’, ‘Won’, and ‘SC1’ denote the unique volatile compounds of the corresponding cultivars. ‘PcS’ and ‘PpS’ indicate the common volatile compounds present in three *P. communis* and three *P. pyrifolia* cultivars. The coordinate unit on the radar map is the quantity of each class of volatile compound **(D, E)** indicate bubble diagrams of esters and alcohols in the ‘common volatile compounds’ and ‘unique volatile compounds’.

Comprehensive analysis was conducted on the volatile profiles of the six cultivars to further elucidate the key volatile compounds contributing to the aroma variations between *P. communis* and *P. pyrifolia* cultivars. As shown in **Figure 3A**, among the volatile compounds found in the three *P. communis* cultivars, 71 were shared by all three cultivars, while 116, 45, and 83 compounds were unique to ‘Rad’, ‘RCF’, and ‘QY’, respectively. Of these shared compounds, 19 exhibited significantly higher levels in the three *P. communis* cultivars than in the three *P. pyrifolia* cultivars, with esters and alcohols being the predominant compounds, accounting for 84% (**Figures 3A, C**; **Supplementary Table S4**). Among the esters and alcohols mentioned above, butyl acetate, hexyl acetate, amyl acetate, ethyl-2-methylbutyrate, butanol, propanol, and 2-methylbutanol have also been found in high concentrations in ‘Conference’, ‘Comice’, and ‘Bartlett’ cultivars, and all have floral and fruity flavors and high OAV values (Makkumrai et al., 2014; Zlatić et al., 2016; Torregrosa et al., 2019), indicating that they play an important role in enhancing the fruit flavor of *P. communis* pear. Among the 116, 45, and 83 unique volatile compounds identified in the ‘Rad’, ‘RCF’, and ‘QY’ cultivars, 33, 13, and 28, respectively, exhibited significantly higher levels compared to the other 5 cultivars (**Figure 3A**; **Supplementary Tables S5–S7**). Esters and alcohols accounted for the highest proportion of these compounds, at approximately 61, 54, and 64% in each cultivar (**Figure 3C**; **Supplementary Tables S5–S7**). Among these esters and alcohols, 1-nonanol, hexenyl acetate, (2z)-, and methyl propionate in ‘Rad’, Hydroxymethyl 2-hydroxy-2-methylpropionate and nerol in ‘RCF’, and 5-hexenyl acetate, isoamyl benzoate, and methyl benzoate in ‘QY’, these volatile compounds not only exhibit high concentrations but also possess floral and fruity flavors, (**Supplementary Tables S5–S7**), indicating they also play a key role in enhancing the aroma of these fruits.

Among the volatile compounds detected in the three *P. pyrifolia* cultivars, 47 compounds were shared by the three varieties, while 50, 35, and 88 compounds were unique to ‘Aki’, ‘Won’, and ‘SC1’, respectively (**Figure 3B**). Of the 47 volatile compounds, only ethyl caproate exhibited a significantly higher concentration in the three *P. pyrifolia* cultivars compared to the three *P. communis* samples (**Figure 3B**; **Supplementary Table S11**). This compound is characterized by its floral and fruity aroma, but the concentration remains relatively low when compared to the high levels of volatile compounds found in the three *P. communis* cultivars. Among the 50, 35, and 88 unique volatile compounds identified in ‘Aki’, ‘Won’, and ‘SC1’ cultivars, 7, 4, and 8, respectively, exhibited significantly higher levels compared to the other 5 cultivars (**Figure 3B**; **Supplementary Tables S8–S10**). Esters and aldehydes constituted the largest proportion. However, their quantities were limited, and most of them were present at low concentrations, consistent with findings in cultivar ‘Hosui’ (Taiti et al., 2017). The limited content and quantity of these compounds was likely the primary factor contributing to the faint fruit aroma of most *P. pyrifolia* cultivars.

As shown above, the quantity of volatile compounds with high contents shared by the three *P. communis* cultivars was greater than that of these present in the three *P. pyrifolia* cultivars. Similarly, the amount of volatile compounds specific to one *P. communis* cultivar at the highest concentration among the six cultivars was significantly higher than that of those unique to one *P. pyrifolia* cultivar. Among the common and unique volatile compounds in the three *P. communis* cultivars, esters and alcohols constituted the largest proportion, and most of them were present at high concentrations (**Figures 3D, E**). Considering that the majority of these alcohols and esters exhibit floral and fruity fragrances (**Supplementary Tables S4–S7**), they were identified as the primary aromatic compounds responsible for the differences between *P. pyrifolia* and *P. pyrifolia* cultivars.

### Synthesis pathways of differential aroma compounds initially elucidated through transcriptomic and non-volatile metabolomic analyses

**Figure 4:**
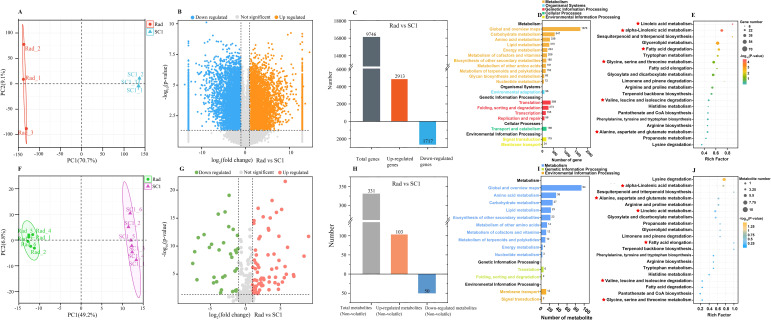
Analysis of the transcriptome and non-volatile compounds of ‘Rad’ and ‘SC1’. **(A)** Principal component analysis (PCA) of genes expressed in ‘Rad’ and ‘SC1’. **(B)** Volcano plot of up- and down-regulated genes (‘Rad’ vs ‘SC1’). **(C)** Histogram of up- and down-regulated genes with KO (‘Rad’ vs ‘SC1’). **(D, E)** KEGG enrichment analysis of up- and down-regulated genes with KO. **(F)** Principal component analysis (PCA) of non-volatile compounds detected in ‘Rad’ and ‘SC1’. **(G)** Volcano plot of up- and down-regulated non-volatile compounds (‘Rad’ vs ‘SC1’). **(H)** Histogram of up- and down-regulated non-volatile compounds with C numbers (‘Rad’ vs ‘SC1’). **(I, J)** KEGG enrichment analysis of up- and down-regulated non-volatile compounds with C numbers.

As previously mentioned, the primary reason for the intense aroma of *P. communis* cultivars was likely attributed to the substantial quantity and high concentration of common and unique esters and alcohols. We selected 16 esters and alcohols from the 19 volatile compounds common to the three *P. communis* cultivars and conducted a preliminary analysis on their biosynthesis pathways (**Table 1**). Our initial objective was to target the synthesis pathways of these 16 esters and alcohols using KEGG pathway enrichment. However, the limited number of volatile compounds included in the KEGG database restricted the effectiveness of pathway enrichment. Considering that volatile compounds are catalyzed by non-volatile substances through enzyme genes, we measured the non-volatile and transcriptomic profiles of ‘Rad’ and ‘SC1’ and compared these data (‘Rad’ vs ‘SC1’) to facilitate enrichment analysis. A total of 31,030 expressed genes and 331 non-volatile compounds were detected in the two varieties (**Supplementary Tables S2, S3**). PCA was conducted on the identified genes and non-volatile compounds (**Figures 4A, F**). The results revealed a distinct separation between the two cultivars, suggesting significant differences in their gene expression profiles and non-volatile compound contents. Further, the biological replicates of each cultivar exhibited consistent clustering (**Figures 4A, F**), indicating the good repeatability of the data. This ensured data quality and satisfied the requirements for subsequent analysis.

**Table 1:** Contents and odor descriptions of 16 volatile esters and alcohols that are common at significantly higher levels in three *P. communis* cultivars than in three *P. pyrifolia* cultivars.

Name	RT^a^	CAS^b^	Contents (ng/g)						Odor^c^	Odor threshold^d^
			Rad	RCF	QY	Aki	Won	SC1		
Hexyl 2-methylbutyrate	17.9805	10032-15-2	14651.25	1608.00	284.10	––––	––––	––––	Apple, Fruit, Green Apple, Strawberry	0.022ug/g (e)
2-Methylbutyl 2-Methylbutyrate	13.7919	2445-78-5	1081.04	858.21	414.03	––––	––––	––––	Apple, Berry, Rum	/
Ethyl-2-methyl butyrate	6.53076	7452-79-1	729.32	1811.83	750.96	347.22	54.08	––––	Apple, Ester, Green Apple, Kiwi, Strawberry	0.011ug/g (e)
Propyl 2-methylbutyrate	9.28241	37064-20-3	1042.49	564.09	5.68	––––	––––	––––	Fruit	0.019ug/g (e)
Isobutyl 2-Methylbutyrate	10.5272	2445-67-2	514.79	204.39	84.92	––––	––––	––––	/	/
N-amyl acetate	10.3782	628-63-7	378.85	91.80	407.29	2.43	23.94	––––	Apple, Banana, Pear	0.043ug/g (e)
N-hexyl acetate	13.5093	142-92-7	20373.86	310.77	7192.09	124.17	118.88	122.11	Apple, Banana, Grass, Herb, Pear	0.002ug/g (e)
N-butyl acetate	7.09138	123-86-4	1197.35	1399.40	5698.55	511.30	746.71	––––	Apple, Banana	0.066ug/g (e)
Ethanol	3.67776	64-17-5	8721.25	5596.28	27140.96	1605.09	1504.02	––––	alcohol	950ug/g (g)
N-propanol	6.227075	71-23-8	13468.83	3017.64	1945.79	––––	––––	––––	Alcohol, Candy, Pungent	9ug/g (e)
N-butyl alcohol	9.59839	71-36-3	11588.30	6793.57	3609.03	22.30	108.37	––––	Fruit	0.5ug/g (e)
Isobutyl alcohol	7.980455	78-83-1	3711.05	652.88	111.36	––––	41.61	––––	Apple, Bitter, Cocoa, Wine	0.033-500 mg/m^3^ (f)
2-Methyl-1-butanol	11.5418	137-32-6	8425.43	575.53	3695.70	––––	––––	––––	Green, Malt, Onion, Wine	0.25ug/g (e)
Citronellol	26.041	106-22-9	3814.20	192.78	16.91	––––	––––	––––	Citrus, Green, Rose	/
Sulcatol	18.8719	1569-60-4	786.13	36.73	59.08	––––	––––	––––	Floral, fruity, vegetable	/
Farnesol, (2z,6e)-	37.3818	3790-71-4	355.93	78.79	32.16	––––	––––	––––	Oil	/

^a^Retention time by DB-Wax column. ^b^CAS number. ^c^Odor descriptor were obtained via the website ‘https://www.femaflavor.org/flavor-library’. ^d^Odor threshold were obtained through: (e) Li, 2012; (f) Van Gemert, 2011; (g) ‘https://mffi.sjtu.edu.cn/database’. ‘––––’indicates that the volatile compounds were not detected by GC–TOFMS. ‘/’ indicates an absence of aroma information associated with the volatile compound.

Among the detected genes, 2913 were up-regulated and assigned KEGG Orthology (referred to as KO), and 1717 were down-regulated and assigned KO. The total number of genes with KO was 9746 (**Figures 4B, C**). Of the detected non-volatile compounds, 103 were up-regulated and had a compound number (referred to as C number), and 50 were down-regulated and had a C number. A total of 331 were non-volatile substances with a C number (**Figures 4G, H**). KEGG enrichment analysis was performed on genes and non-volatile compounds with KO and C numbers. These differentially expressed genes and non-volatile compounds were primarily enriched in pathways related to carbohydrate, amino acid, lipid, energy metabolism, and secondary metabolite biosynthesis. Their sub-pathways included glycine, serine, and threonine metabolism, valine, leucine, and isoleucine degradation, linoleic acid and alpha-linolenic acid metabolism, and fatty acid degradation (**Figures 4D, E, I, J**). Due to their role in providing intermediate compounds for aromatic compound synthesis, these sub-pathways were selected as the primary sources for the production of 16 esters and alcohols.

### Further identification of the synthesis pathways for differential aroma compounds

**Figure 5:**
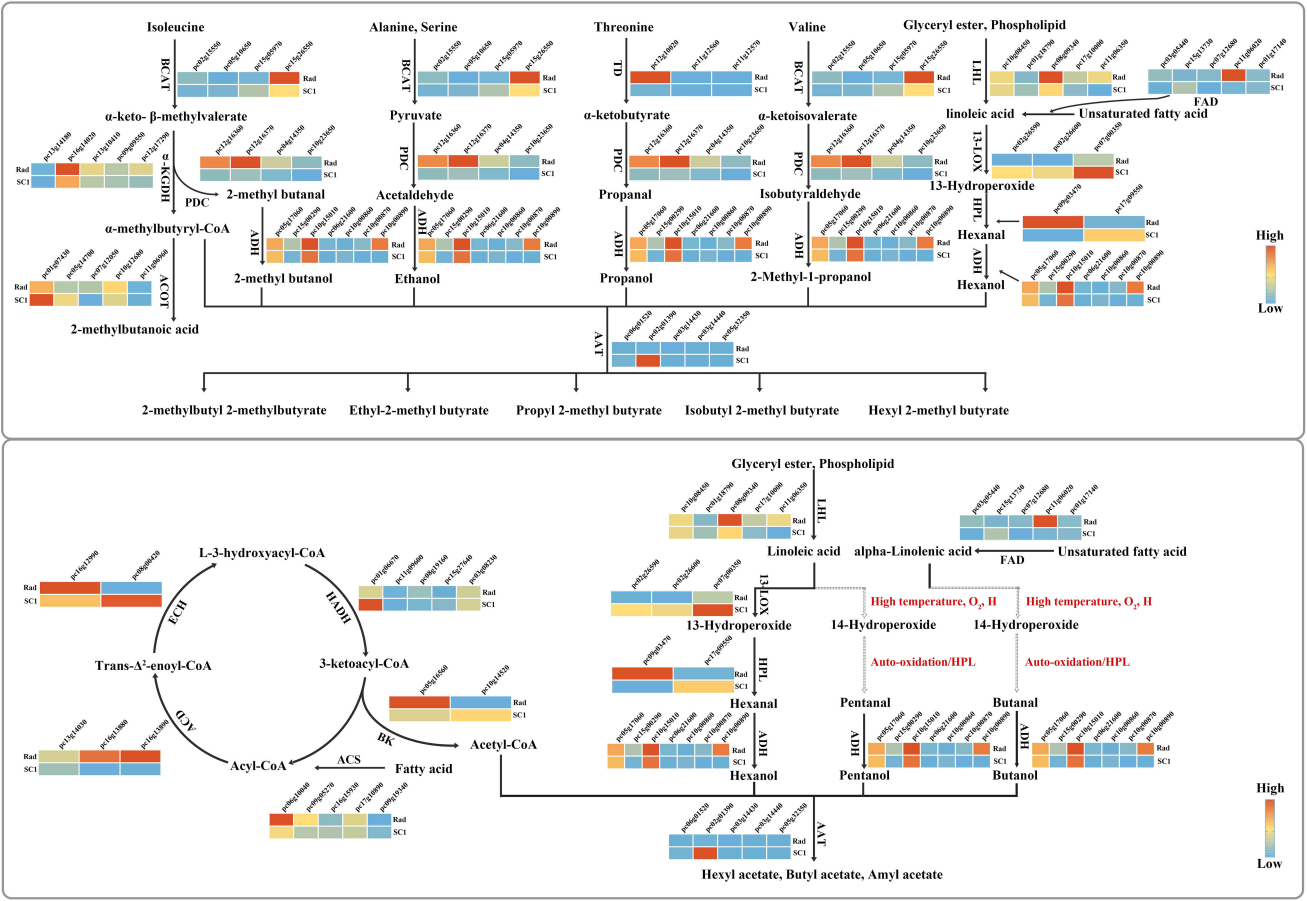
Potential synthesis pathways of 16 esters and alcohols. For simplification, ‘pycom’ in gene nomenclature is abbreviated as ‘pc’. The solid arrows indicate that each step of the reaction has been documented. The dashed arrows suggest that the reaction steps are not explicitly recorded and are inferred based on the existing literature.The following are the full names of the key enzymes. BCAT, Branched-chain amino acid aminotransferase; α-KGDH, α-Ketoglutarate dehydrogenase; ACOT, Acyl-CoA thioeaterase; PDC, Pyruvate decarboxylase; ADH, Alcohol dehydrogenase; AAT, Alcohol acetyltransferase; TD, Threonine dehydratase; LHL, Lipid hydrolase; FAD, Fatty acid desaturase; 13-LOX, 13-Lipoxygenase; HPL, Hydroperoxide lyase; ACS, Acyl-CoA Synthetase; ACD, acyl-CoA dehydrogenase; ECH, Enoyl-CoA hydratase; HADH, Hydroxyacyl-CoA dehydrogenase; BK,Beta-ketothiolase.

Ester compounds are synthesized through the reaction of acyl-CoA with alcohol, which is catalyzed by alcohol acyltransferase (AAT) (Liu et al., 2023). Among the 16 esters and alcohols, acyl-CoA can be categorized into two groups: 2-methylbutyryl-CoA and acetyl-CoA. According to the KEGG enrichment results (**Figures 4E, J**), 2-methylbutyryl CoA was likely derived from isoleucine degradation, while acetyl CoA was probably produced by the degradation of saturated fatty acids. In these two approaches, the enzyme genes responsible for catalyzing each step in the reaction exhibited higher expression levels. In particular, the genes for fatty acyl CoA synthetase (pycom09g05270), acyl CoA dehydrogenase (pycom16g13880), enoyl CoA hydratase (pycom16g12990), acyl CoA thiolase (pycom05g16560), and branched amino acid tranminase (pycom15g26550) showed significantly higher expression levels in ‘Rad’ than in ‘SC1’ (**Figure 5**). Alcohols that undergo reactions with two types of fatty acyl CoA and those that were detected independently were categorized into two groups. The first group consisted of straight-chain alcohols, including ethanol, propanol, butanol, pentanol, and hexanol. The second category comprised branched-chain alcohols, such as 2-methylbutanol, 2-methylpropanol, and citronellol. Based on the KEGG enrichment analysis (**Figures 4E, J**) and the existing literature, ethanol might originate from alanine degradation, while propanol might primarily result from threonine dehydration (Wang et al., 2002). The expression levels of key enzyme genes involved in these two degradation processes, including branched amino acid tranminase (pycom15g26550), threonine dehydratase (pycom12g10020), and pyruvate decarboxylase (pycom12g16360, pycom10g23650), were markedly elevated in ‘Rad’ compared to ‘SC1’ (**Figure 5**). Hexanol has been proposed to be synthesized via the LOX pathway of linoleic acid (Scala et al., 2013). Key enzymes in this pathway included lipases (encoded by pycom08g09340 and pycom11g06350), pyruvate decarboxylases (encoded by pycom12g16360, pycom10g23650), and alcohol dehydrogenases (encoded by pycom10g00870 and pycom10g00890). The expression levels of these genes were higher in ‘Rad’ than in ‘SC1’ (**Figure 5**).

Currently, the fatty acid pathway, particularly the LOX pathway, is a focal point in pear fruit aroma research (Shi et al., 2019; Li et al., 2022). This study revealed that, in addition to the fatty acid pathway, some amino acid degradation processes, especially threonine dehydration, which has been rarely documented, also played a significant role in aroma synthesis in *P. communis* cultivars. These results offer valuable information into the synthesis pathways of other aromatic compounds in pear fruit.

### Identification of key enzyme genes in synthesis pathways

**Figure 6:**
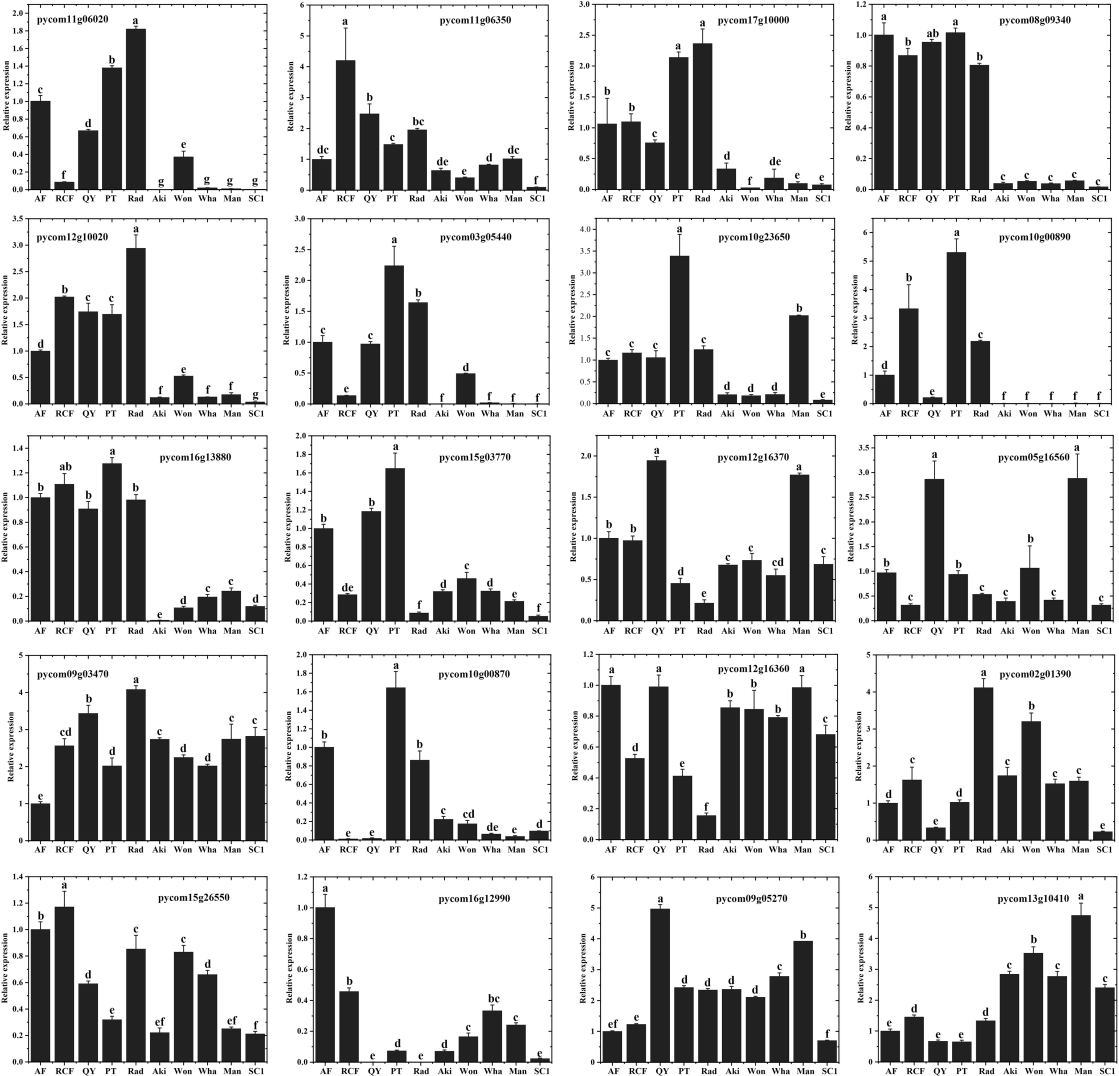
Expression of 20 key enzyme genes in the synthesis pathway across five *P. communis* and five *P. pyrifolia* cultivars. The error bars were calculated based on standard deviation. Different letters indicate significant differences at level p value = 0.05.

To identify the key enzyme genes involved in the synthesis pathways of 16 esters and alcohols, we selected 20 enzyme genes from the synthesis pathway shown in **Figure 5**, which showed significantly higher expression in ‘Rad’ than in ‘SC1’, and analyzed their expression levels in five *P. communis* and five *P. pyrifolia* cultivars. Subsequently, we performed RT-qPCR analysis on these 20 genes. The results demonstrated that the amplification efficiency of each primer pair ranged from 90% to 109% (**Supplementary Table S14**), which was consistent with the findings of Dai et al. (2023) and Yao (2023). This indicated that the PCR conditions, including the quality and concentration of the cDNA, were well optimized and satisfied the requirements for subsequent gene quantification analysis. The relative expression levels of genes were quantified using the 2^ –ΔΔCt method as described by Livak and Schmittgen (2001). As shown in **Figure 6**, the expression levels of nine genes, namely, *PcTD* (*pycom12g10020*), *PcACD* (*pycom16g13880*), *PcPDC* (*pycom10g23650*), *PcADH* (*pycom10g00890*), *PcLHL* (*pycom08g09340*, *pycom11g06350*, *pycom17g10000*), and *PcFAD* (*pycom11g06020, pycom03g05440*), were significantly higher in the five *P. communis* cultivars than in the five *P. pyrifolia* cultivars. There were no significant differences in the expression levels of the remaining eleven genes between the five *P. communis* and five *P. pyrifolia* cultivars (**Figure 6**). This suggests that the nine genes play a crucial role in the synthesis of 16 esters and alcohols in *P. communis* cultivars, and their high expression might constitute a primary factor leading to the disparity in aromatic compounds between the *P. communis* and *P. pyrifolia* cultivars.

## Discussion

### Speculation on the synthesis of butanol and pentanol in volatile compounds

Based on the current literature, the synthesis pathways for butanol and pentanol remain undetermined and can only be hypothesized through existing analysis and research. There are three primary methods for alcohol synthesis in plants. First, amino acids are catalyzed to form alcohols via pyruvate decarboxylase (PDC) (Song et al., 2015). Second, fatty acyl CoA is catalyzed by acyl CoA reductase to produce alcohols (Napora-Wijata et al., 2014). Lastly, unsaturated fatty acids generate alcohols through the LOX pathway (Scala et al., 2013). Amino acids containing a greater number of carbon atoms typically undergo decarboxylation to form alcohols with functional groups, which deviate from straight-chain alcohols, such as butanol and pentanol. The expression of annotated acyl-CoA reductase genes was not detected in ‘Rad’ or ‘SC1’, suggesting that butanol and pentanol synthesis via acyl-CoA reductase was highly improbable (genes encoding acyl-CoA reductase were detected in the pear genome annotation file, https://www.rosaceae.org). Unsaturated fatty acids, including linoleic acid and alpha-linolenic acid, are catalyzed by 9-LOX or 13-LOX to form 9-hydroperoxides (9-ROOH) or 13-hydroperoxides (13-ROOH) after the loss of hydrogen atoms at C11. These hydroperoxides subsequently undergo cleavage to produce C6 alcohols or C9 enols (Buescher and Buescher, 2001; Scala et al., 2013).

Prior studies have indicated that hydrogen atoms adjacent to the C=C double bond are more prone to abstraction compared to those directly on the C=C double bond (Porter et al., 1995), and hydrogen atoms adjacent to either side of the C=C double bond are likely to be abstracted (Cao et al., 2020). Therefore, in linoleic acid and α-linolenic acid, in addition to hydrogen atoms at C11, hydrogen atoms at C14 are prone to loss. This phenomenon has been documented by Feussner and Kühn (2000). However, there are limited reports of enzymes that catalyze the formation of 12-ROOH or 16-ROOH following the loss of hydrogen atoms at C14. There is also a lack of information regarding this in the pear genome annotation file (https://www.rosaceae.org). Recent studies have indicated that under high-temperature conditions, the hydrogen atoms at positions C8 and C11, which are bonded to the C=C double bond in oleic acid, can undergo dehydrogenation (Cao et al., 2020). These molecules undergo rearrangement and react with oxygen to form 8-ROOH, 10-ROOH, 11-ROOH, and 9-ROOH. This process leads to the formation of octanol, nonyl alcohol, and decanol through cleavage reactions (Cao et al., 2020). Therefore, we further hypothesize that the hydrogen atoms at the C14 position of linoleic acid are lost during the high-temperature extraction phase of HS-SPME. C14-ROOH and C12-ROOH were generated following rearrangement and subsequent oxygenation, with the former potentially decomposing to form pentanol. Similarly, following the loss of hydrogen atoms at the C14 position of α-linolenic acid, a series of rearrangements and oxygenations led to the formation of C14-ROOH, C12-ROOH, and C16-ROOH. C14-ROOH might decompose to yield butanol (**Figure 5**).

### The role of highly expressed key genes in enhancing the aroma content of *P. communis* cultivar fruit

*PcACD* (*pycom16g13880*) encodes acyl-CoA dehydrogenase, a crucial enzyme involved in isoleucine degradation and fatty acid β-oxidation (Maggio-Hall et al., 2008). In this study, the elevated expression of this gene in *P. communis* cultivars compared to *P. pyrifolia* cultivars might result in increased 2-methylbutanoyl-CoA and acetyl-CoA production, leading to 2-methylbutyryl and acetyl ester formation. *PcTD* (*pycom12g10020*) encodes threonine dehydratase, which catalyzes the conversion of threonine to α-ketobutyric acid. This intermediate is subsequently decarboxylated and dehydrogenated to form propanol (Wang et al., 2002). The high expression of this gene in the *P. communis* cultivars could account for the higher levels of propanol and 2-methylbutyrate propanol compared to those in the *P. pyrifolia* cultivars. *PcLHL (pycom08g09340*, *pycom11g06350*, *pycom17g10000*) encodes lipid hydrolases, of which *pycom08g09340* encodes monoacylglycerol lipase, *pycom17g10000* encodes triacylglycerol lipase, and *pycom11g06350* encodes phospholipase. All three enzymes catalyze the release of fatty acids via hydrolysis reactions (Zimmermann et al., 2004; Scala et al., 2013; Grabner et al., 2017). The high expression levels of the three genes in the *P. communis* cultivars might contribute to increased fatty acid production, thereby providing essential precursors for the synthesis of numerous alcohols and esters. *PcFAD* (*pycom11g06020*, *pycom03g05440*) encodes fatty acid desaturases and catalyzes unsaturated fatty acid production (Somerville and Browse, 1996). The elevated expression of these genes in the *P. communis* cultivars might result in an increased content of unsaturated fatty acids, which can indirectly influence the formation of alcohols through the LOX pathway. *PcPDC* (*pycom10g23650*) and *PcADH* (*pycom10g00890*) encode pyruvate decarboxylase and alcohol dehydrogenase, respectively. These genes are positioned at the terminal position of the aroma synthesis pathway (Wang et al., 2019; Manríquez et al., 2006). The high expression levels in *P. communis* cultivars might have a direct impact on the final alcohol compound concentration.

In prior research on the aroma of pear fruits, *LOX*, *HPL*, *PDC*, *ADH*, and *AAT* were determined to be the primary structural genes in elucidating the aroma synthesis mechanism in pear fruit (Scala et al., 2013; Manríquez et al., 2006). This study found that, in addition to the aforementioned genes, threonine dehydratase *PcTD* (*pycom12g10020*), monoacylglycerol lipase (*pycom08g09340*), and triacylglycerol lipase (*pycom17g10000*) might also play crucial roles in aroma synthesis in *P. communis* cultivars. These findings not only expand the repertoire of significant structural genes involved in pear aroma synthesis pathways, but also provide novel target sites for enhancing the pear aroma content through gene editing. Furthermore, while ketones, acids, and other compounds constitute only a minor fraction of volatile compounds, certain high-concentration components such as 2-octanone, heptyl ketone, n-butyric acid, and n-undecane are noteworthy. Many of these compounds possess fruity aromatic properties (**Supplementary Tables S5–S10**). Therefore, future aroma research should also focus on these components.

## Conclusion

**Figure 7:**
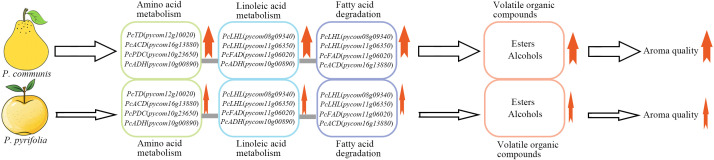
Potential formation model of differential aroma compounds between *P. communis* and *P. pyrifolia* cultivars. Bold red arrows adjacent to the green, blue, and purple oval frames represent a high level of expression of key genes that are crucial for the synthesis of differential aroma compounds. The thick red arrow next to the orange oval frame indicates a potential increase in the ester and alcohol concentrations. The bold red arrow situated at the base of the upper row indicates an enhancement in the aromatic quality of *P. communis* cultivar fruit. The hollow thick arrows denote the significant roles of elevated expression levels of key genes and increased ester and alcohol concentrations in enhancing fruit aroma. Thin red arrows indicate low expression levels and concentrations of genes and metabolites within the oval frame. The hollow and thin arrows indicate low expression of key genes, and reduced levels of esters and alcohols have a minimal influence on the development of fruit aroma. The thin red arrow at the base of the row indicates a diminished aromatic quality in *P. pyrifolia* cultivar fruit.

Through the analysis and discussion of the aforementioned results, it was concluded that 2-methylbutyrate (2-methylhexyl butyrate and 3-methylbutyl 2-methylbutyrate), acetate (butyl acetate, hexyl acetate, and amyl acetate), and straight-chain alcohols (ethanol, butanol, propanol, and 2-methylbutanol) are likely the primary volatile compounds responsible for the differences in fruit aroma between *P. communis* and *P. pyrifolia* cultivars. These compounds might enhance their content primarily through the up-regulation of key enzyme genes in fatty acid and amino acid metabolism pathways, such as the enzyme genes identified in this research, including monoacylglycerol lipase, threonine deaminase, and long-chain acyl-CoA dehydrogenase. The elevated levels of these volatile compounds and the up-regulated expression of key genes might result in high aroma quality in *P. communis* cultivars compared to *P. pyrifolia* cultivars (**Figure 7**).

## Data Availability

The datasets presented in this study can be found in online repositories. The names of the repository/repositories and accession number(s) can be found in the article/**Supplementary Material**.
